# Characterization of the water bodies of Extremadura (SW Spain)

**DOI:** 10.1007/s10661-023-11187-9

**Published:** 2023-04-13

**Authors:** Mohamed Amine Abdennour, J. Francisco Lavado Contador, Jesús Barrena González, Chiara Piccini, Anthony Gabourel Landaverde, Manuel Pulido Fernández

**Affiliations:** 1 Grupo de Investigación Geoambiental, Instituto Universitario de Investigación Para El Desarrollo Territorial Sostenible (INTERRA), Universidad de Extremadura, 10071 Cáceres, Spain; 2 Council for Agricultural Research and Economics, Research Centre for Agriculture and Environment, CREA-AA), Via Della Navicella 2-4, 00184 Rome, Italy

**Keywords:** Reservoirs, Agriculture, Livestock, Spatial analysis, Land use

## Abstract

Extremadura is the region that stores the greatest amount of fresh water in Spain. Such water is mainly used for power generation, irrigation in agriculture, biodiversity conservation, tourism, recreation, and human and livestock consumption. Nevertheless, crucial information on the total number of water bodies and their geometrical characteristics and spatial distribution patterns are still missing. Thus, our main goal was to characterize the Extremenian water bodies geometrically and spatially through different statistical techniques such as kernel density, Moran’s index, the Getis-Ord Gi*, and principal component analysis (PCA). Firstly, all existing hydrological information was gathered, and using aerial aircraft imagery and satellite images, each water body (WB) was then carefully collected, checked, and corrected. We have inventoried 100,614 WBs (mean density: 2.45 WB km^2^), irregularly distributed on the territory. WBs with an area < 0.01 km^2^ (100 ha) represent 64.5% of the total. A multivariate statistical study was conducted, showing that livestock, aridity of the climate, and topography are the main factors controlling the density of water bodies (WBs) in this area. It can be concluded that monitoring of small bodies is crucial to understand their spatial distribution, since they are spread over areas in which extensive farming and commercial crops such as tobacco strongly influence the way of living of many families.

## Introduction

Water is an essential resource for life and a paramount issue for ecological security and socioeconomic development, particularly in geographical areas where water is a limiting factor during almost the whole year (e.g., arid and semi-arid regions) (Balist et al., 2022) or in particular periods of hydrological deficit. This is the case of the Mediterranean environment during summertime, drought periods, or in particular cultivation cycles, mainly due to its characteristic irregular rainfall patterns (Fernández & Schnabel, 2010). Such periods of rainfall scarcity usually correspond to much higher evaporation rates and extra needs for human use, like agriculture irrigation and livestock consumption. Therefore, the design and construction of ponds, dams, and reservoirs have been one of the most recognizable strategies performed under these irregular climate conditions (Pulido Fernández et al., 2019).

The importance of storing water has not gone unnoticed by the scientific community. In fact, artificial water bodies (WBs) are present in all biogeographical regions, from the desert to the tundra in the Arctic Circle (Céréghino et al., 2014). One of the main research topics regards stored water and human use, but also, the role of water bodies on biodiversity (Bichsel et al., 2016), their contribution to ecosystem services (Holgerson & Raymond, 2016; Tallis & Polasky, 2009), the sensitivity to climate change (Minga-León et al., 2018), and the vulnerability to anthropogenic disturbances (Lischeid et al., 2018; Marín-Comitre et al., 2022) are relevant (Biggs et al., 2017).

Water bodies are often the most productive ecosystems, as they have a high diversity of flora and fauna (Kelly-Quinn et al., 2017). They play a very important role in socioeconomic conditions as they are used for commercial fish farming, for animal consumption, and for irrigating small plots. Small water is essential for natural flood regulation, trapping sediments and contaminants, retaining nutrients and conserving biological diversity, and extending to downstream rivers, lakes and estuaries (Riley et al., 2018). Identification of WBs using modern techniques, such as aerial imagery and geographic information systems, allows local managers to lead and engage in a plan against water contamination and to protect water to support farmers and livestock needs, which will certainly contribute to the socioeconomic development of this region.

Some interesting works have been published regarding water availability, estimating water volume if the total surface covered by water is known. McDonald et al. (2012) performed this kind of study in the USA, while Marín-Comitre et al. (2021) surveyed easily identifiable small water bodies (SWBs) in Spain, accurately delineated from free aerial images using Geographic Information System (GIS) tools, by digitizing (WBs) and then extracting them as a layer on ARCGIS from an orthophoto, through using Extract By Mask tool. Estimating the storable water using GIS and remote sensing can be a feasible solution for small areas such as a single farm (< 1000 ha) or municipality (< 100 km^2^) (Abijith et al., 2020; Al-Khuzaie et al., 2020; Duarte et al., 2014; Rashash & El-Nahry, 2015; Saranya & Saravanan, 2020). Technical limitations arise when the target of the study is a large land surface area such as, for instance, the region of Extremadura (41,634 km^2^) (Terasmaa et al., 2019). An accurate delimitation of the WBs, even of the bigger ones, can be problematic (Tymków et al., 2019), since the hydrological network provided by topographic maps is not perfectly accurate, and digitizing missing water bodies turns quite laborious.

Monitoring WBs on a regular basis in large geographical regions such as Extremadura requires a large amount of human capital and tools, which are often not easy to find for land managers and health organizations (Sivanpillai & Miller, 2010). WBs can be monitored and mapped using remote sensing imagery with middle or high spatial precision. For example, Landsat Satellite Mapper (TM) data has a spatial precision of 30 m, which limits its applicability for the identification and mapping of small water bodies. Aiming to avoid confusion with other classes and to identify SWBs, aerial images with a very high spatial precision (25 cm) are required for accurate mapping. Today’s aerial imaging, as well as geospatial data processing and visualization equipment, provides a multipurpose ability. An aerial image gives a great opportunity for analyzing surface spatial elements such as soil, vegetation, urban areas, and hydrography.

The reasons for choosing the Extremadura region as study area are twofold: (i) Extremadura contains about 30% of the dammed fresh water in Spain (Pulido et al., 2019); and (ii) the dominant land use corresponds to agriculture, as one of the main regional income sources. Commercial crops, fruit orchards, olive groves, vineyards, and extensive farming are widespread over the region (Jaraíz-Cabanillas et al., 2018; Morant et al., 2020). Regarding livestock, in Extremadura, the agro-silvo-pastoral Dehesa system occupies most of the area, and the highest number of sheep and farms dedicated to lamb meat production is present (Thomasz et al., 2020). Extensive farming, corresponding to 58% of the land surface of Extremadura, is strongly influenced by water scarcity, with a direct effect both on pasture production (Díaz et al., 2018) and on water storage (Fernández & Schnabel, 2010). In addition, the average increase observed in grazing intensity entails problems of land degradation (Pulido et al., 2018), negatively affecting also water quality (Marín-Comitre et al., 2022). Particularly important as a trend along last decades is the fact that thousands of SWBs have been created by livestock farmers in the region—mainly since 1970s (Pulido et al., 2020)—used as watering ponds for livestock. The impact of such abundant SWBs on the water cycle, including the amount of stored water, is still misunderstood, and knowledge is lacking.

Indeed, an important research question arises about the size of the WBs (Komarkova et al., 2018). It is well-known that SWBs are usually better suited to support freshwater species, in some contexts, than rivers or large lakes (Oertli et al., 2009). According to Terasmaa et al. (2019), even less diverse SWBs (e.g., bog pools) often support unique plant and animal species and contribute better to the habitat diversity. Mendonça et al. (2017) concluded that SWBs, particularly agricultural ponds, can sequester more organic carbon per unit area than larger WBs. In this study area, the positive or negative effects of SWBs were not investigated yet, being still necessary to undertake a description of their abundance, geometrical characteristics, and spatial distribution, hence contributing to improve our knowledge about the amount of stored water and its distribution over the region. A similar study could help also to provide advice for farmers about local adaptations to climate change.

The main goal of this study was to undertake the characterization of the size and spatial distribution of the SWBs of Extremadura using GIS, remote sensing, orthophotos, and statistical and spatial analysis, being aware of the existing uncertainty about the abundance of SWBs at regional scale and of their relevance over key elements of the water cycle and the ecosystems and knowing also that their number increases in a sustained temporal trend.

## Materials and methods

### Study area

**Fig. 1:**
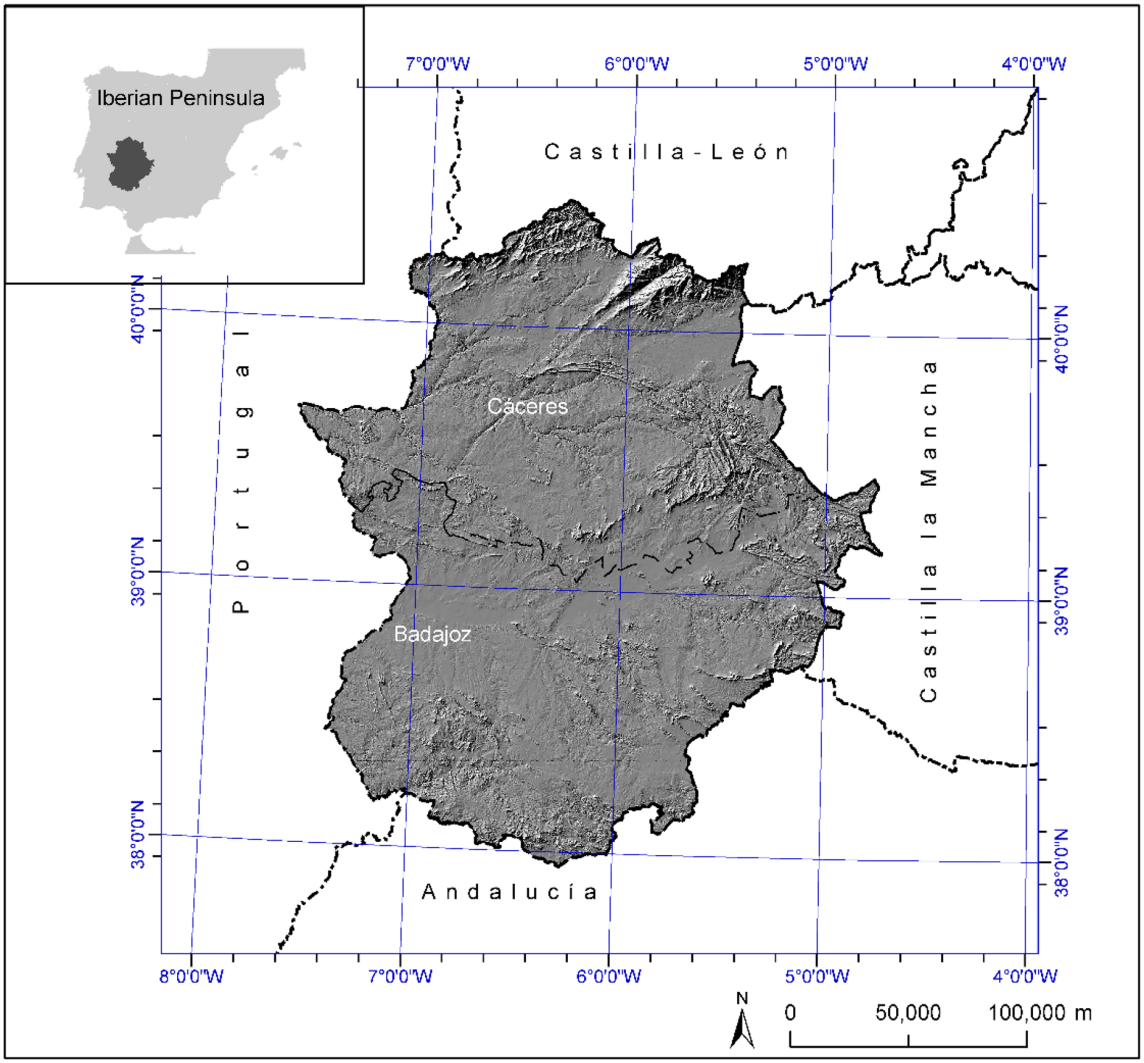
Location of the study area

Extremadura is a region of Spain, located in the central-west part of the Iberian Peninsula, surrounded by the regions of Castilla y León, Castilla-La Mancha, and Andalucía in the north, east, and south, respectively, and by Portugal in the west. Its surface area is 41,364 km^2^ (8.2% of the whole country) and comprises two provinces: Cáceres and Badajoz (Fig. 1). About 60% of the territory is covered by wooded rangelands, known as dehesas, and natural grasslands mainly devoted to extensive farming, being the remaining surface mostly occupied by agricultural lands and mountainous areas. The mean altitude is 425 m, ranging from 45 m of the Guadiana river valley to more than 2000 m in the northern mountain ranges. Six landscape dominions can be distinguished in the region: mountains, piedmonts, sierras, peneplains, river plains, and steep river banks. The official population of the region is 1.065 million people (2% of Spanish population), distributed in 388 municipalities, with an average density of approx. 25 inhabitants/km^2^. Extremadura is considered as a typical rural region, since more than 25% of the population lives in settlements of less than 10,000 inhabitants, and 30% of the active population works in the primary sector: agriculture and livestock (Instituto Nacional de Estadística, 2022).

**Fig. 2:**
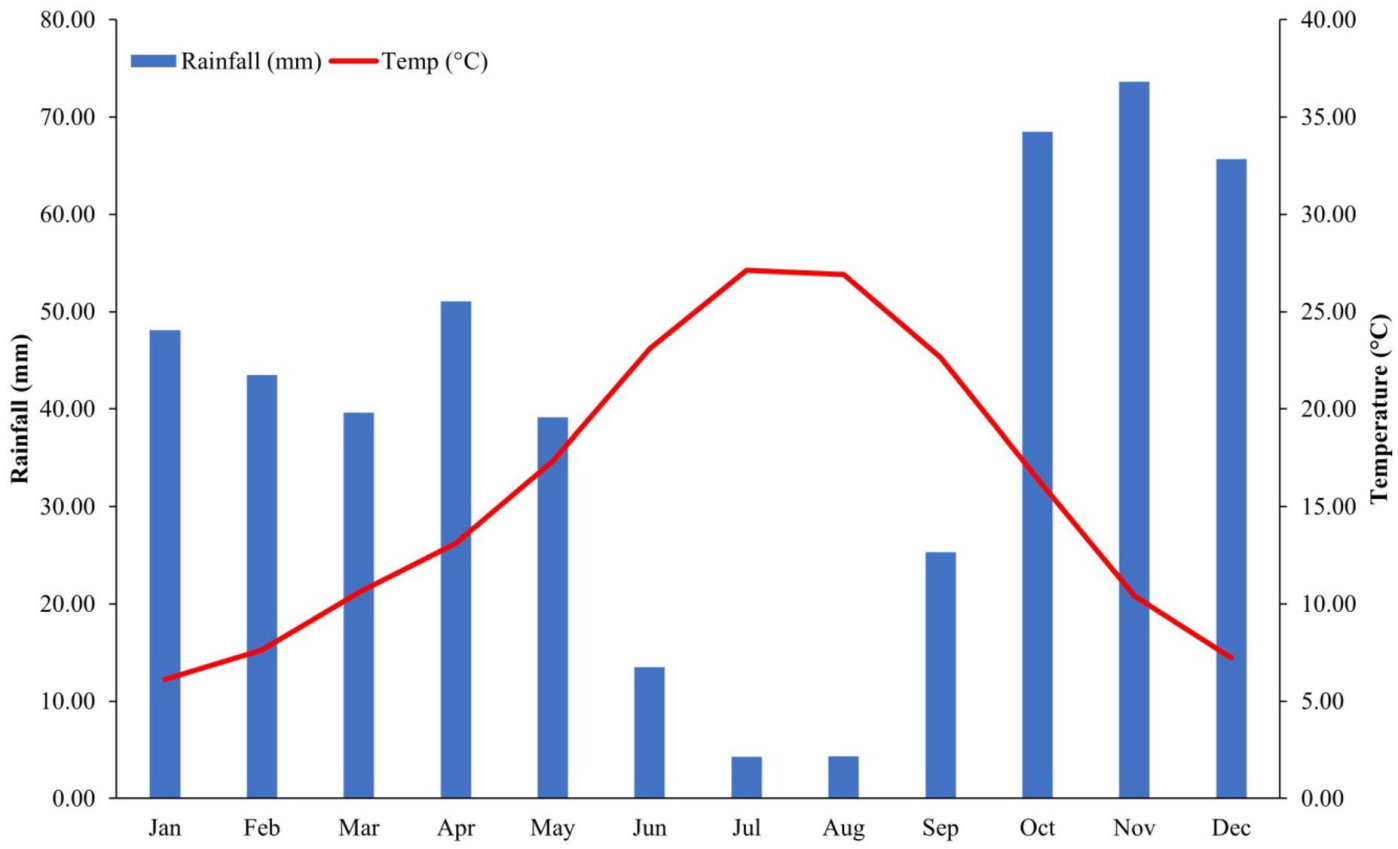
Climograph generated with meteorological data from Caceres station (period: 1981–2021)

The dominant climate is Mediterranean, with dry and hot summers, mild winters, and highly variable rainfall patterns that mostly occur from October to May (Moral et al., 2016). The climograph (Fig. 2) shows the average precipitation and temperature for the Cáceres meteorological station data set (1981–2021).

### Land classification

**Fig. 3:**
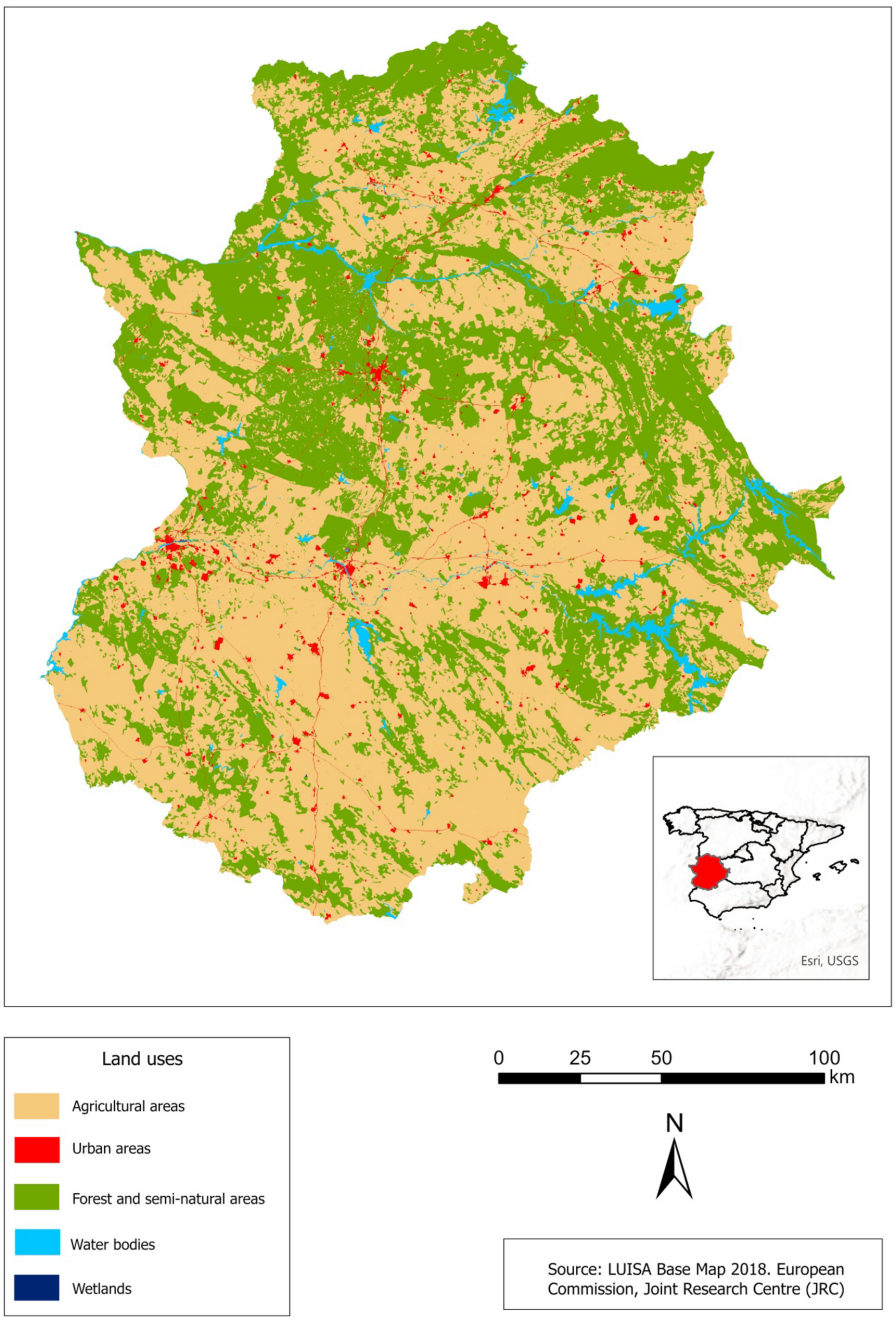
Land cover of the study area

In order to define the major land cover classes in this area, we have used the LUISA 2018 base map (Land Use-based Integrated Sustainability Assessment), which is a high-resolution land use and cover map developed and produced by the European Commission’s Joint Research Centre (Pigaiani & e Silva, 2021). It corresponds to a modified and improved version of the CORINE Land Cover 2018 map. Then, to check if the water bodies have a privileged trend of a certain class, different land use classes were identified in the study area, in particular urban area, agricultural area, forest and semi natural area, wetlands, and class of water. Figure 3 shows land use maps for the Extremadura region.

### Experimental design and workflow

This research was primarily aimed at characterizing the size and the spatial patterns of the SWBs in Extremadura.

It must be considered that, at present, there does not exist any complete hydrological map or data source that incorporates all the WBs for the studied region, neither big water masses as large dams and reservoirs nor smaller ones as livestock watering ponds or other SWBs used for a variety of purposes as agricultural irrigation at local scales. Such SWBs are highly abundant, are very dynamic in time, and are usually underestimated in official cartographies and databases. Therefore, in order to build the cartographic database used as main data source for this study, it was necessary to collect a variety of incomplete primary data sources (initial source data in Table 1), each of them partially incorporating SWB data.

**Table 1:** Primary incomplete sources used to build the small water body map used in this study

**Source**	**Scale/spatial resolution**	**Source**
Topographic Cartography of Extremadura	1:10,000	http://sitex.gobex.es/SITEX/centrodescargas
COPERNICUS Riparian zones	1:20,000	https://www.copernicus.eu/en
National Topographic Database	1:25,000	https://centrodedescargas.cnig.es/CentroDescargas/catalogo.do?Serie=CAANE#
SIGPAC	1:5000	http://sitex.gobex.es/SITEX/centrodescargas/view/11
PNOA	25 cm of pixel size	https://centrodedescargas.cnig.es/CentroDescargas/index.jsp

**Fig. 4:**
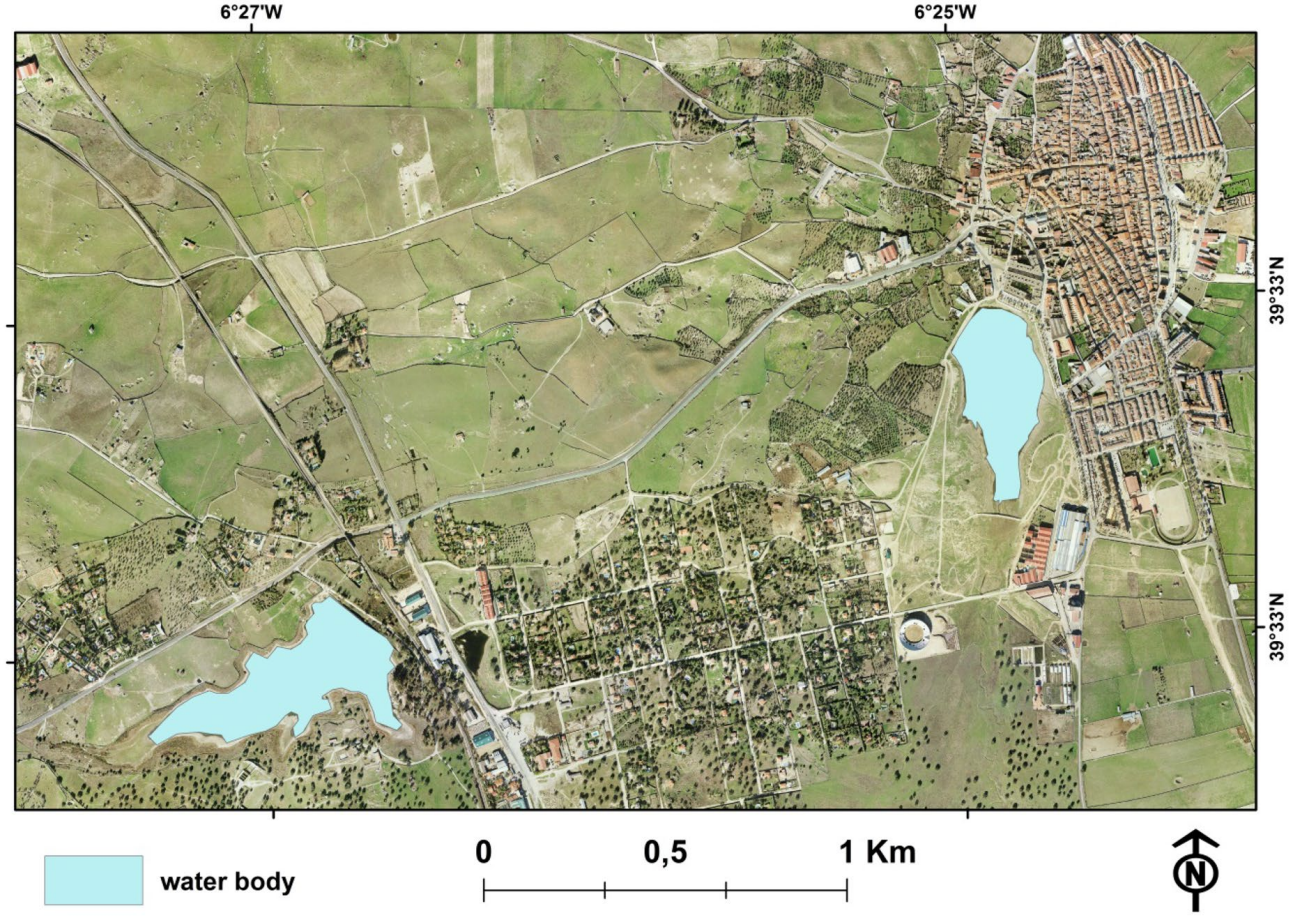
Example of water bodies digitized from an orthophoto image

In order to determine the spatial distribution of the WBs existing in Extremadura, a single map was created using ArcGIS software from the combination of hydrological information, the 1:10,000 topographic map of Extremadura, orthophotographs—historical orthophotographs of the Plan Nacional de Ortofotografía Aérea (PNOA) and the current maximum orthophotography of the PNOA—and ponds and reservoirs of Sistema de Información Geográfica de Parcelas Agrícolas (SIGPAC). Through the use of the most recent orthophotographs with a resolution of 25 cm, Fig. 4 shows how two water bodies, located in the Casar de Caceres area in the center of the study area, were digitized as polygons in ArcGIS. For the surface calculation of each water body, the command “Calculate Geometry Attributes” in ArcGIS was used.

GIS tools were then used to spatially correct, remove duplicates, complete the polygons, join elements, and merge different data sources. Most of the SWBs were checked, and their polygons were corrected until reaching the maximum discernable surface occupied by water. A number of missing WBs to be added to the final map were also digitized, using ortophotographs of the year 2019 provided by the Plan Nacional de Ortofotografía Aérea (PNOA) with 25 cm of spatial resolution.

Table 1 shows the consulted data sources, incorporating partial SWB information. Finally, a map was created with all the SWBs of Extremadura extracted from such primary data sources.

From the whole number of WBs (several thousands), the final number of SWBs for the study was selected based on several criteria: (i) size; (ii) use in livestock, and (iii) the possible accuracy in digitizing the WB, because small water bodies are used in livestock, and they are the subject of our study; their digitization was carried out with high accurate for precision mapping.

SWBs were classified by size in five classes (the class classification is in Table 2) proposed by Meybeck (1995). The patterns of spatial distribution were characterized with a kernel density estimator (Lin et al., 2011; Spencer et al., 2017) and other local spatial statistics such as semivariogram modeling (Abdennour et al., 2020; Goovaerts, 2001; Juan et al., 2011; Mahmood & Batool, 2020) and Getis-Ord Gi* and Moran’s I values (Liu et al., 2022).

**Table 2:** Geometrical parameters of the considered small water bodies by size class. Total values are shown

**Class**	**Size (km**^**2**^**)**	**Count**	**%**	**Area (km**^**2**^**)**	**%**	**Perimeter (km)**	**%**
5	>0.1	35	0.03	5.92	7.26	75.49	0.93
4	0.01–0.1	937	0.93	23.02	28.21	693.09	8.54
3	0.001–0.01	13,928	13.84	31.92	39.12	2653.25	32.70
2	0.0001–0.001	53,942	53.61	19.10	23.41	3824.25	47.13
1	<0.0001	31,772	31.58	1.63	2.00	867.84	10.70
Total		100,614	100.00	81.59	100.00	8113.92	100.00

### Size classification

As said above, WBs were classified according to Meybeck (1995), who collected the number of WBs per area in the world and established 5 categories by a factor of 10 between them (Bartout et al., 2015): (1) < 0.0001 km^2^ (< 100 m^2^), (2) 0.0001–0.001 km^2^ (100–1000 m^2^), (3) 0.001–0.01 km^2^ (1000–10,000 m^2^), (4) 0.01–0.1 km^2^ (10,000–100,000 m^2^), and (5) > 0.1 km^2^ (> 100,000 m^2^). The following parameters were calculated for each size class: total count, area, and perimeter. The same classification system has been already used in other countries such as France, Finland, and Estonia (Terasmaa et al., 2019). Results have been discussed and compared with other works at larger scales.

### Spatial analysis

The pattern of concentration of SWBs over the region was studied by using both a kernel density estimator (Hart & Zandbergen, 2014; Victoriano & Lacatan, 2020) and values of Getis-Ord General Gi* and Moran’s Index, all of them embedded in the software ArcGIS v.10.0 (ESRI, 2010). Kernel density estimation is a data smoothing technique to transform a set of point observations (i.e., the centroides of each SWB) into a continuous surface indicating the density of the individual observations in space (Kloog et al., 2009), where points located near the center of the kernel are assigned a weighting factor higher than those located near the edge (Bonaiti & Fipps, 2016). The Getis-Ord Gi* statistic is widely used to perform hotspot analysis, providing information about the aggregation degree of a spatial variable in terms of high-value areas (hot spots) and low-value areas (cold spots) (Chambers, 2020; Kumari & Pandey, 2020). The Moran’s I provides an estimation of the spatial autocorrelation of the variable under interest, where values close to 0 are considered as randomly distributed, and, conversely, values close to 1 show a concentrated pattern.

### Geostatistical analysis

Geostatistics allows to verify the spatial structure of a variable and to define such structure by using a semivariogram (or simply variogram) function (Abdennour et al., 2020; Bradaï et al., 2016). In our case, it is useful to check if each model actually follows the surface areas of the WBs. Therefore, the first step was the definition of the spatial variation by building a variogram. In this study, the geostatistical analysis was carried out by performing the variogram that represents the semivariance of the difference between attribute values for all points separated by a lag distance (Piccini et al., 2014). Based on Eq. (1) of Delhomme (1978):

1 $$\gamma \left(h\right)=\frac{1}{2N\left(h\right)}{\sum }_{i=1}^{N\left(h\right)}{\left[Z\left({x}_{i}\right)-\left(Z\left({x}_{i}+h\right)\right)\right]}^{2}$$

where γ (*h*) is the experimental semivariance value for all pairs separated by a distance *h* (lag); *Z* (${x}_{i}$) is the value of the considered variable in each point; *Z* (${x}_{i}+h$) is the value of the variable in points at a discrete distance *h*; ${x}_{i}$represents the position where each *Z* (${x}_{i}$) value was measured; and *N*(*h*) represents the number of pairs of observations at a distance *h*.

Plotting all the semivariances versus their distances, a variogram cloud is produced, and, averaging the values for the lag distance, the experimental variogram is obtained. The semivariances are typically smaller at shorter distance and may reach an upper bound (sill) at a finite distance (range), beyond which there is no longer spatial autocorrelation (Marchetti et al., 2012; Oliver & Webster, 2015). The nugget variance, a positive intercept on the ordinate, is an uncorrelated component indicating short distance variation, which includes measurement error, sampling error, inter-sample error, and unexplained and inherent variability.

A mathematical model is then fitted to the experimental variogram (Goovaerts, 2001) to minimize the variance of the errors. The spatial dependence of the data can be classified based on the nugget/sill ratio (%). A ratio < 25% indicates high spatial dependence, a ratio of 25–75% indicates moderate spatial dependence, and a ratio > 75% indicates low spatial dependence (Abdennour et al., 2019; Arslan, 2012; Bradaï et al., 2016; Cambardella et al., 1994). Low values can be interpreted as a concentration pattern.

### Influencing variables

Aiming at understanding which variables control or explain the spatial distribution of SWBs in the region, a multivariate analysis was used in this study. A principal component analysis (PCA) was performed to reduce the complexity of a newly developed dataset that integrates a large amount of information, including physical and socioeconomic factors that could contribute to control the SWB construction and therefore their spatial patterns in the region. Eleven parameters were calculated and developed as maps under ArcGIS: (i) the SWBs density (WB); (ii) the drainage density (DD); (iii) the population density (PD); (iv) the livestock density (LD); (v) the elevation (Elev); (vi) the slope (slope); (vii) the precipitation (rain); (viii) the land surface temperature (LS-temp); (ix) the soil moisture (SM); (x) the topographic wetness index (TWI); and (xi) the standard precipitation index (SPI).

All the 388 municipalities of the Extremadura region were chosen as the spatial foundation for this dataset, and their spatial extent was used for extracting representative values (total values or mean ones) of the eleven variables selected by manual GIS procedures. To obtain or calculate the necessary data, publicly available Digital Elevation Models (DEM) and aircraft imagery, Landsat 8 TOA (top-of-atmosphere reflectance) satellite images of 30-m-resolution, and raster maps from climate engine platform were used.

In this research, we used data obtained from the free web application Climate Engine (http://ClimateEngine.org), which uses Google’s parallel cloud computing platform Google Earth Engine (Gorelick et al., 2017) to allow users to process, visualize, download, and share various global and regional climate and remote sensing datasets and products (e.g. Tagged Image File Format (TIFF) and time series) in real time (Huntington et al., 2017).

Raster maps for rain, LS-T, SM, and SPI were downloaded from the climate engine platform, after which the value of each WB was extracted in ArcGIS, using the Extract Multi Values to Point option in the spatial analysis tool. Elev, slope, DD, and TWI were calculated from the topographic map of the study area. For each municipality, we calculated and extracted the representative value of each parameter used in this study, calculated by spatial analyst tool in ArcGIS.

## Results

### Spatial distribution of SWBs by size

Table 2 shows the calculated geometrical parameters of the SWBs by size class: count, area, perimeter, and relative abundance (%). A total amount of 100,614 WBs were inventoried. Overall, they occupy a land surface of 81.59 km^2^, showing a total perimeter of 8113.92 km. Considering their abundance by class, the dominant one was class 2 (regular ponds: 100–1000 m^2^) with more than half (53.61%) of the total SWBs, followed by the small ponds (class 1: < 100 m^2^). Considering the covered area, class 3 (big ponds: 1000–10,000 m^2^) was the dominant class (39.12%); nevertheless, class 2 (regular ponds: 100–1,000 m^2^) reached the highest percentage in terms of total perimeter (47.13%).

Class 5 (big reservoirs, size > 100,000 m^2^) comprised only 35 WBs, covering almost 6 km^2^ in area and more than 75 km in perimeter. In other words, only 0.03% of the considered SWBs occupy 7.26% of the total area covered by water. Class 4 (10,000–100,000 m^2^) reached higher values than class 5 for all the parameters, representing the dominant reservoir type (small reservoirs). In class 1 (very small ponds, < 100 m^2^) 31,772 WBs are present, representing only 2.00% of the total area and 10.70% of the total perimeter. Nonetheless, they reached a higher total perimeter than classes 4 + 5.

**Fig. 5:**
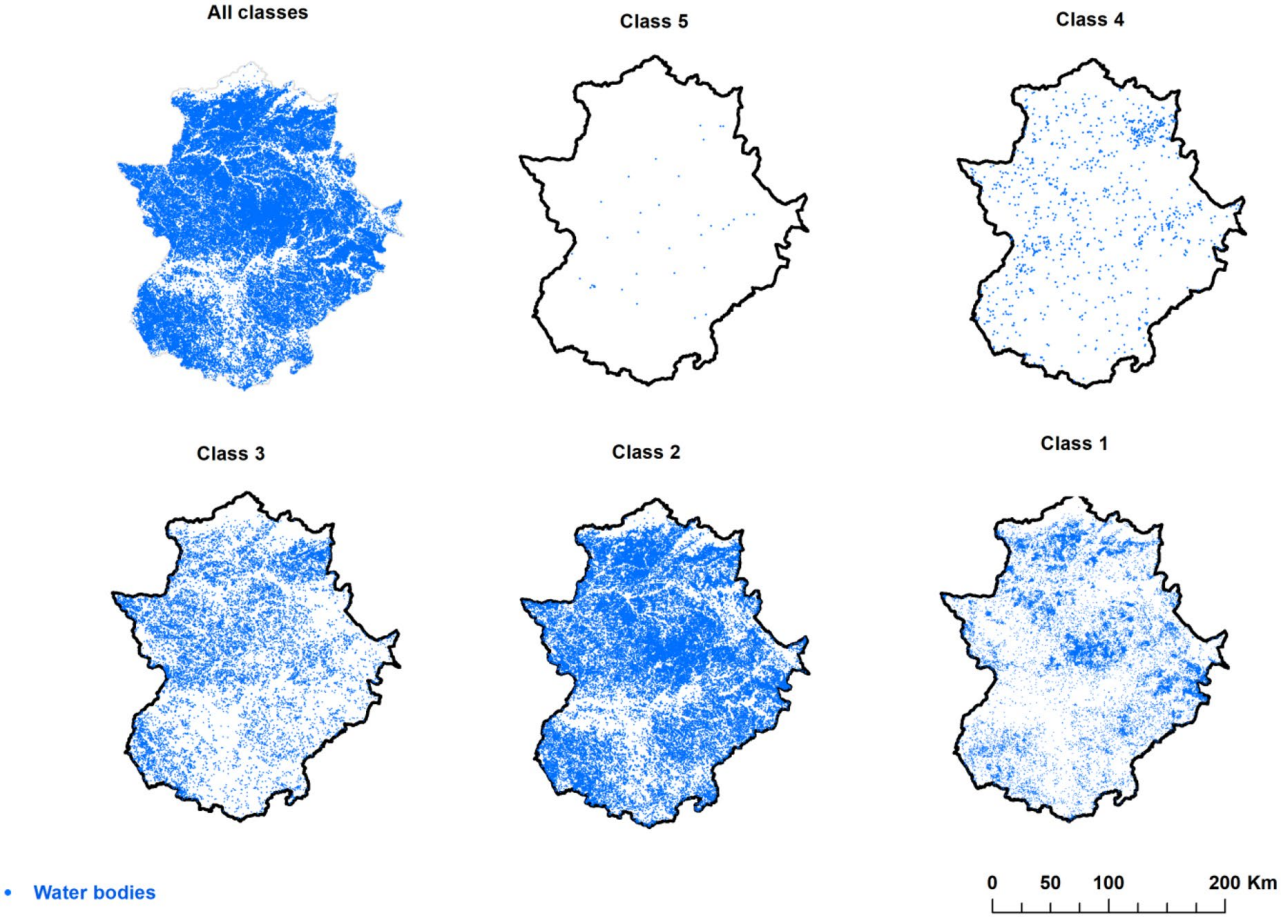
Spatial distribution of water bodies of Extremadura by size classes

Figure 5 shows the spatial distribution of each class size throughout Extremadura. SWBs are abundant around the region, except in the highest mountains and near the main rivers. Big reservoirs (class 5) are distributed in a sort of strip in a southwest-northeast direction. They are mostly reservoir built to dam the water of the most important rivers: Guadiana and Tagus. From the class 4 to the class 2 (including class 3), a progressive increase in water bodies density is observed, but a similar and homogeneous distribution. Finally, the class 1 (small ponds) showed higher densities in some spatial clusters that are easily recognizable: north and south-east.

**Fig. 6:**
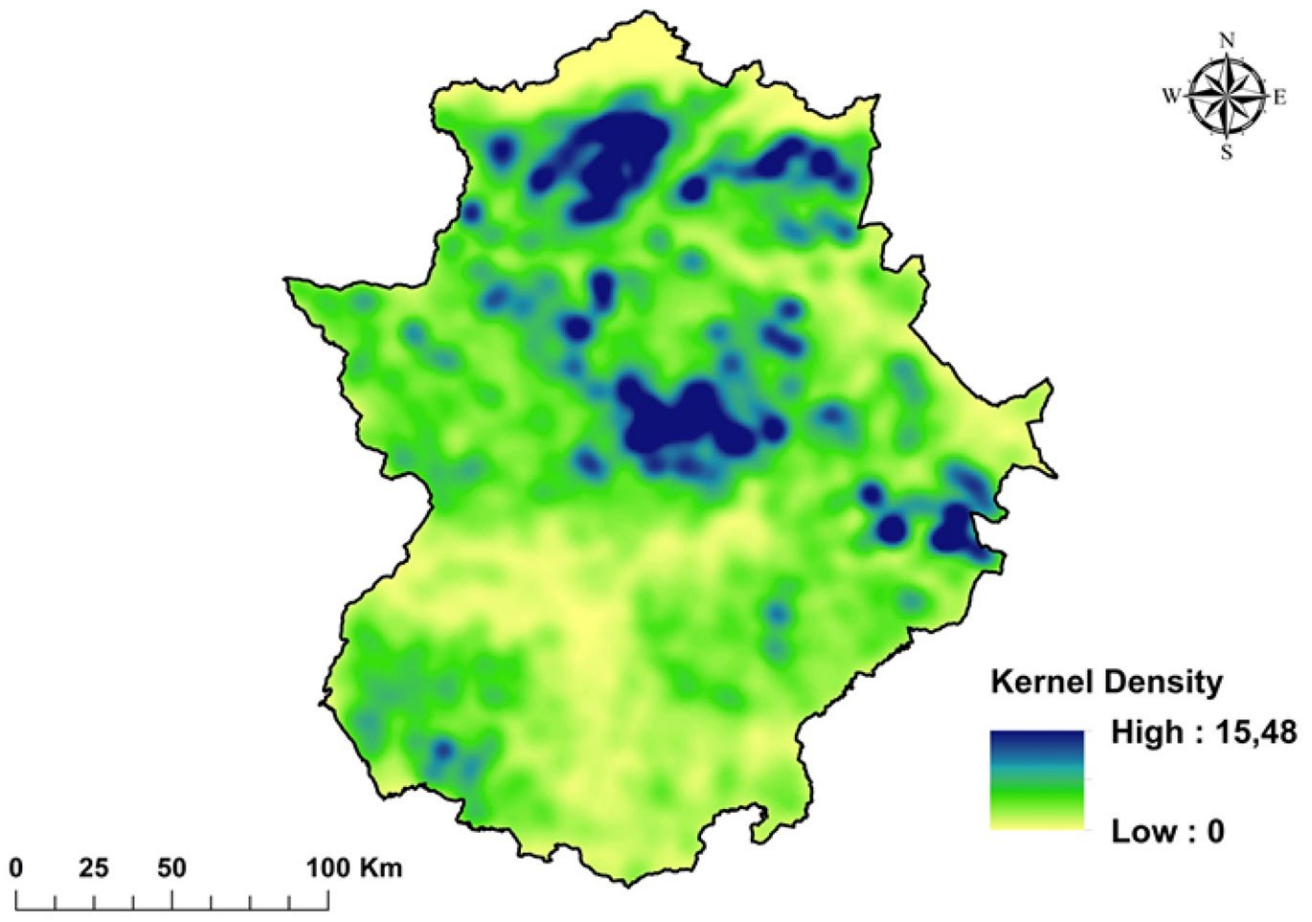
Map of kernel density of the water bodies in Extremadura

The nearest neighbor analysis returned a ratio of 0.62 m (*p* < 0.000, *z*-score =  −228.7), i.e., a value less than 1, confirming a concentration trend or clustering of the elements. This trend is also confirmed by the mean distance among WBs—248.05 m, much lower than the expected mean distance of 398.11 m, that can be considered the minimum distance for a random distribution. Moreover, the map of the kernel density (Fig. 6) shows the areas in which the SWBs are highly concentrated (> 10 SWBs per km^−2^). Four main areas can be observed where SWBs are highly aggregated: two of them are located in the north, where small parcels of irrigated lands with tobacco and maize plantations are frequent, and the other two are located in the center and at the south-east of the region, where low tree density or treeless pasturelands are abundant.

### Moran’s I analysis

**Fig. 7:**
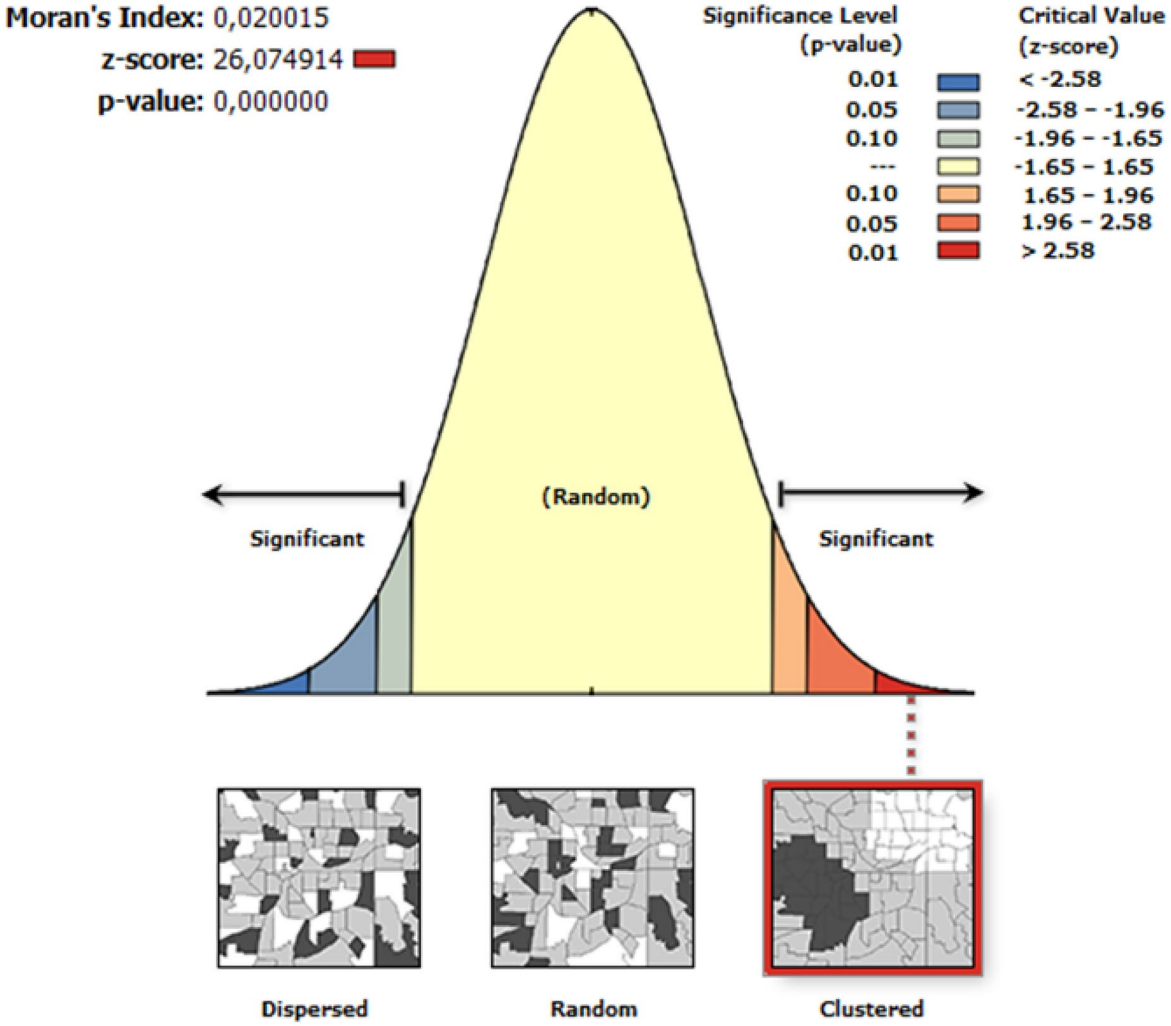
Moran’s index report for the water bodies of Extremadura

The Moran’s I coefficient was positive (0.020), indicating that the data are spatially autocorrelated (clustered). The *z*-score was 26.07, and the *p*-value was 0.000, suggesting a likelihood of random pattern lower than 1% (Fig. 7). Figure 4 shows the visual report of this index as provided by the ESRI ArcGIS v. 10.0 software.

### Hot and cold spot analysis

**Fig. 8:**
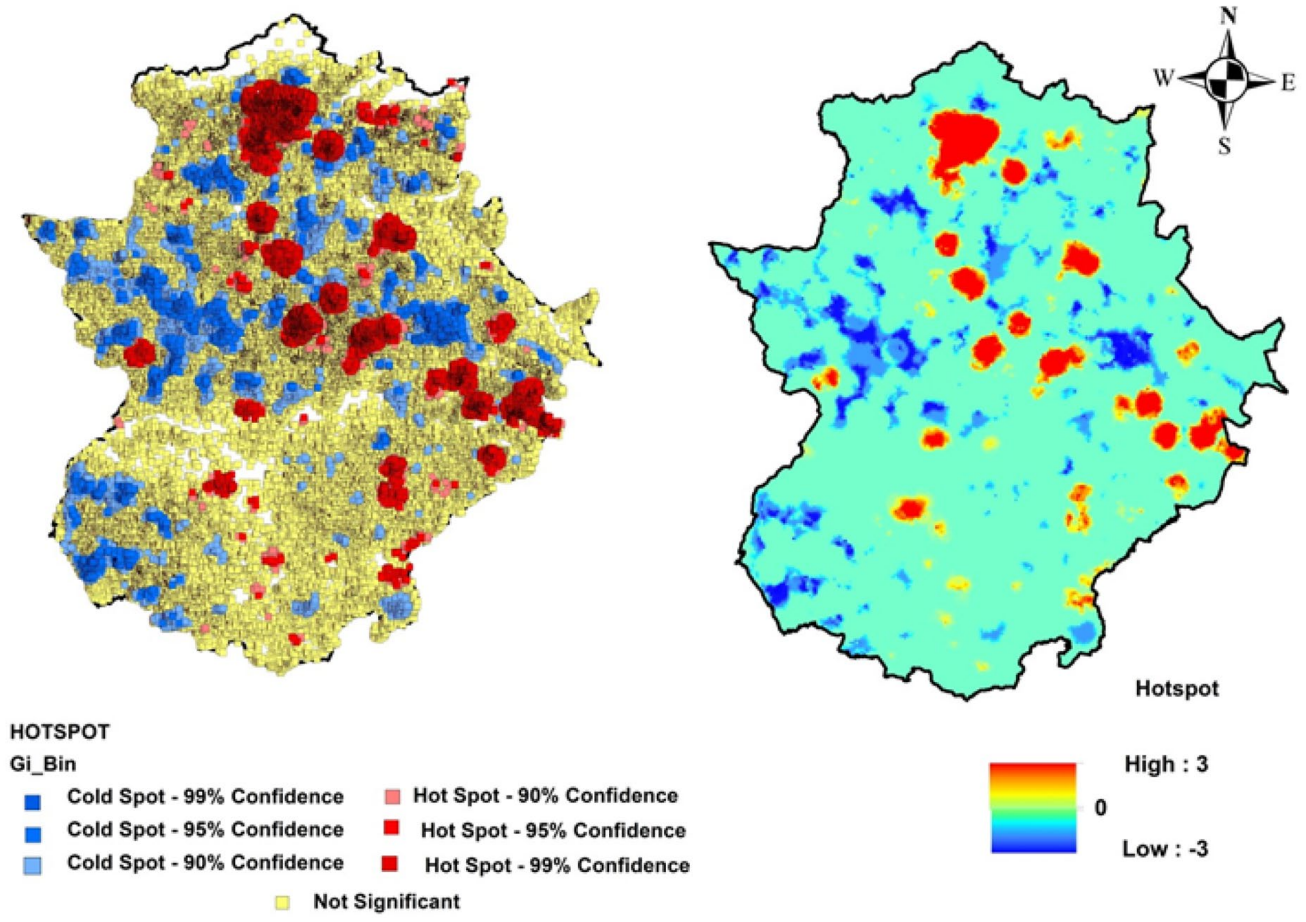
Hot and cold spot analysis of the water bodies in Extremadura

The hot and cold spot analysis was performed over the selected SWBs in Extremadura through a Getis-Ord Gi* statistics; in Fig. 8, hot and cold spots are differentiated at 99%, 95%, and 90% confidence levels, with 0 indicating no statistical significance. The hot areas where statistical significance was obtained are red colored, and cold ones are blue colored, while the yellow-colored areas have no statistical significance. This analysis confirms the patterns already showed by the kernel density analysis, with a sort of spatial stripe going from northwest to southeast, more relevant in the northern part of the region (province of Cáceres).

### Geostatistical analysis

**Fig. 9:**
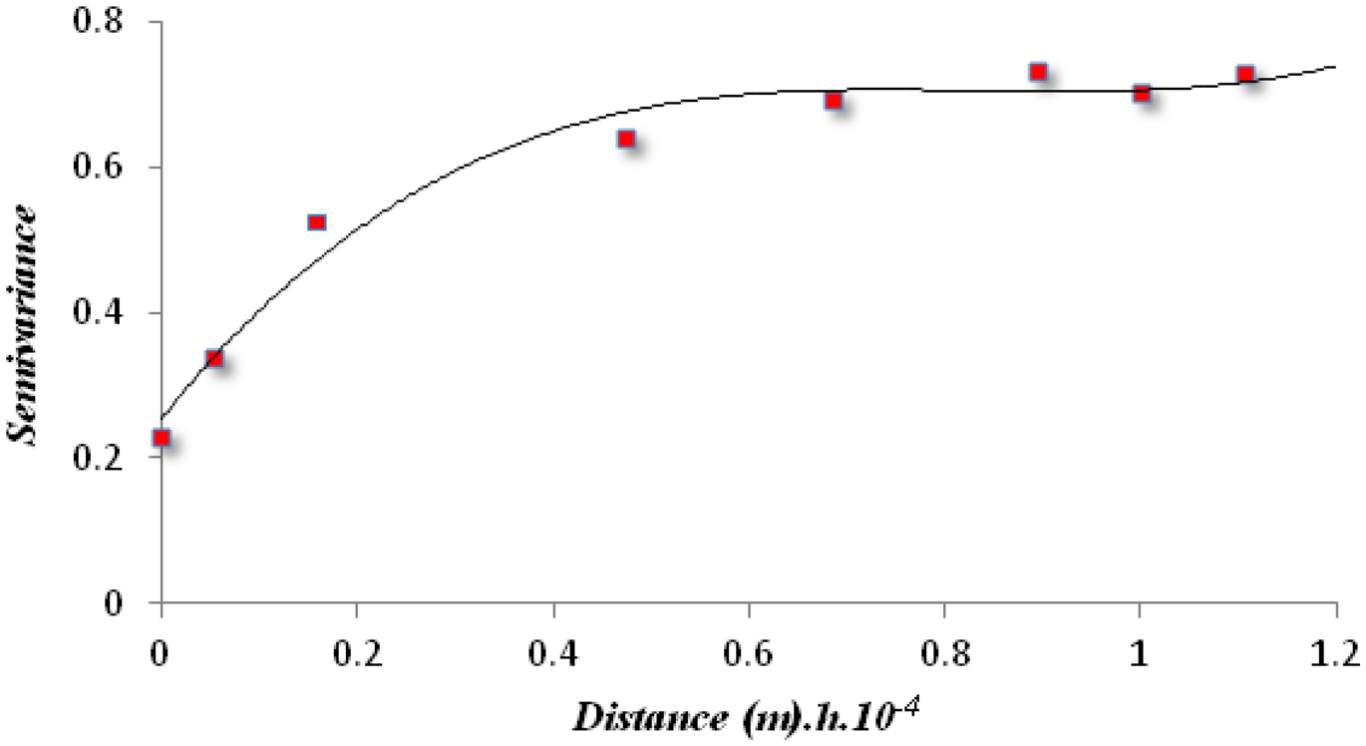
Omnidirectional variogram of water body sizes

Figure 9 shows the variogram theoretical model that best fitted our data, i.e., the spherical model. The nugget effect was 0.23. The sill was 0.75, and the range was 5060 m. The value of the nugget/sill ratio was 30.6% that can be interpreted as a moderate spatial dependence among the SWBs. The variogram clearly shows that the spatial distribution of the size of the considered WBs follows a defined spatial structure and is not random—probably, some contextual physical or socioeconomic variables lead the design and construction of the SWBs in the region.

### Results of the principal component analysis to understand influencing variables

Aiming to facilitate a consistent evaluation of all the variables used in this study and to better understand which parameters control and explain the density and spatial distribution of SWBs, a multivariate analysis was used. A PCA was used to reduce the complexity of the original data, converting them into new uncorrelated variables called principal components (PCs), which are a linear combination of the original variables appearing in a decreasing order of importance.

The descriptive statistics for the 11 considered PCs, analyzed over the 388 municipalities, is presented in Table 3. The values of the standard deviation for some parameters (rain, elevation, and population density) show a large variability due to the different climate, topography, and the socioeconomic conditions of the study area.

**Table 3:** Descriptive statistics of the considered parameters

	**Count**	**Mean**	**SD**	**Min**	**25%**	**50%**	**75%**	**Max**
WB	388	2.90	2.81	0.02	1.02	1.95	3.80	19.85
LD	388	0.32	0.28	0.00	0.12	0.27	0.44	2.56
Elevation	388	444.93	182.51	177.00	313.00	409.50	514.00	1226.00
DD	388	1.60	0.40	0.30	1.35	1.58	1.83	2.96
LS-Temp	388	30.21	3.48	16.48	28.06	30.50	32.73	37.95
Rain	388	576.67	216.83	319.18	408.73	490.47	678.11	1260.58
Slope	388	7.85	6.47	0.36	3.56	5.57	9.99	37.34
SPI	388	−0.04	0.41	−0.93	−0.36	−0.04	0.19	0.93
TWI	388	6.62	1.57	3.91	5.60	6.31	7.42	13.95
PD	388	27.77	54.93	1.03	7.08	14.03	28.49	782.19
SM	388	27.00	3.25	22.30	24.41	26.36	29.47	34.62

*WB *density of SWBs, *LD* livestock density, *DD* drainage density, *LS-Temp* land surface temperature, *SPI* standard precipitation index, *TWI* topographic wetness index, *PD* population density, *SM* soil moisture. *SD* standard deviation, *Min* minimum, *Max* maximum, 25%, 50%, and 75%, percentiles

A Pearson’s correlation coefficient “r” (significant at *p* < 0.001) was calculated, to show the quantitative relationships among the different variables. The correlation values among the eleven parameters are showed in Table 4. It can be observed that LD, SPI, and slope show a moderate to low correlation with SWBs density.

**Table 4:** Pearson’s correlation coefficient among the 11 parameters selected to assess SWB density in the municipalities

	**WB**	**DD**	**PD**	**LD**	**Elev**	**Slope**	**Rain**	**LS-temp**	**SM**	**SPI**
DD	0.08									
PD	–0.08	0.23								
LD	0.33	–0.01	–0.06							
Elev	–0.16	–0.54	–0.16	–0.16						
Slope	–0.19	–0.32	–0.04	–0.19	0.56					
Rain	–0.03	–0.31	–0.10	–0.28	0.52	0.43				
LS-temp	–0.00	0.18	0.002	0.3	–0.45	–0.48	–0.51			
SM	0.02	–0.35	–0.10	–0.30	0.45	0.38	0.79	–0.51		
SPI	0.26	0.1	0.001	0.05	–0.10	0.02	0.60	–0.23	0.53	
TWI	0.14	0.08	0.04	0.04	–0.25	–0.51	–0.10	0.09	–0.12	0.06

*WB* density of SWBs, *LD* livestock density, *DD* drainage density, *LS-temp* land surface temperature, *SPI* standard precipitation index, *TWI* topographic wetness index, *PD* population density, *SM* soil moisture, *Elev* elevation

By using the PCA, the collinearity among the variables is reduced, while preserving the maximum amount of information in a smaller number of dimensions. Therefore, PCA is used herein to better understand the factors that lead SWB construction in the studied region (their mean density in the municipalities).

**Fig. 10:**
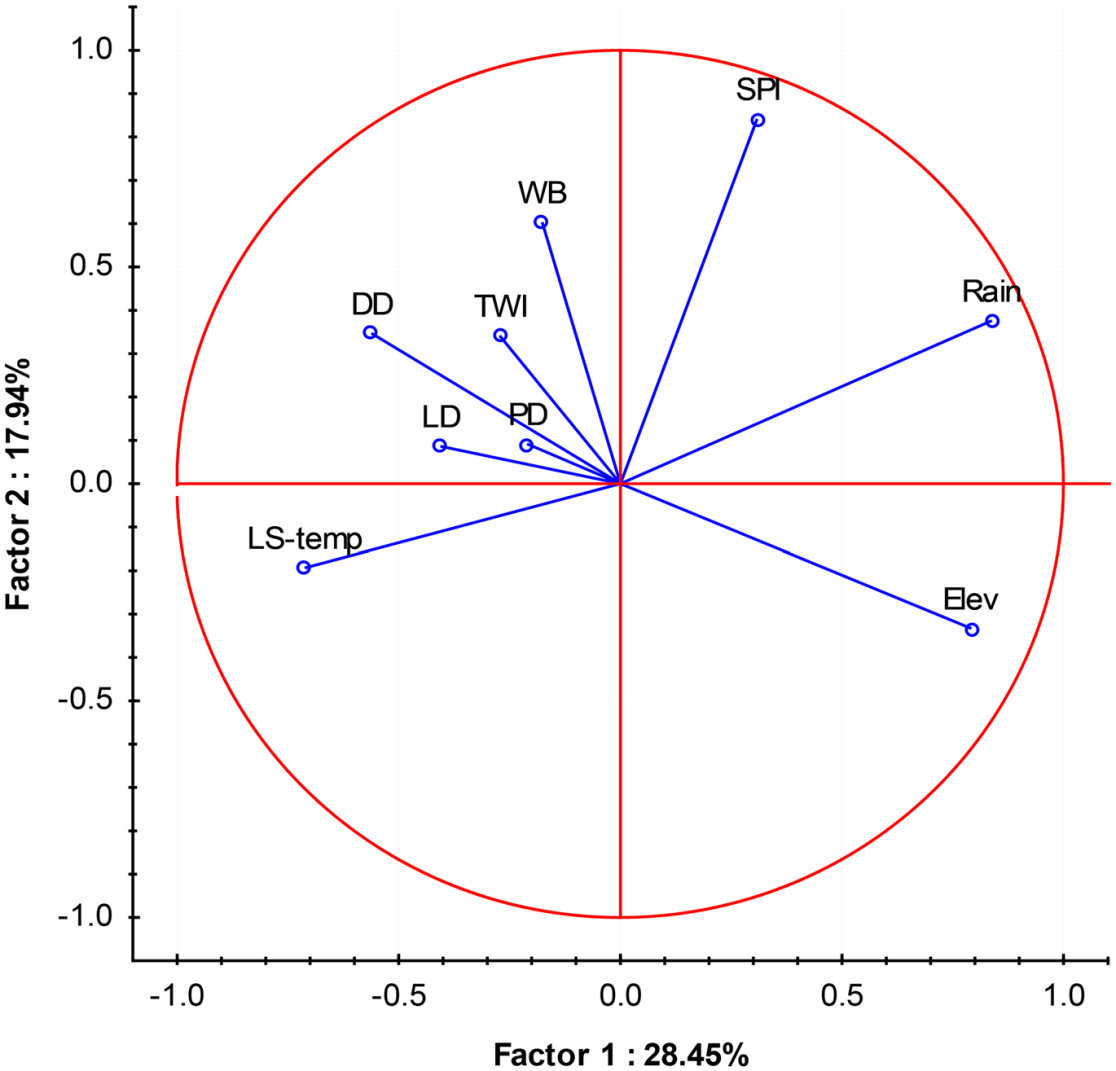
Projection of the variables on the plane defined by factors 1 and 2. WB, density of SWBs; LD, livestock density; DD, drainage density; LS-temp, land surface temperature; SPI, standard precipitation index; TWI, topographic wetness index; PD, population density; Elev, elevation

In a classical PCA, a higher eigenvalue means that the resulting PCs give a greater contribution to explain the variation in the original data. In the PCA, the first four PCs explained 72% of the total variance in the data matrix, and parameters showing loadings beyond ±0.50 and eigenvalues approaching or exceeding 1 are considered significant. As the main result, physical factors explain most of the variance of the first two PCs, related to climate and topography, that mainly control the SWBs density over the study area. An important role was played also by some socioeconomic aspects, as population and livestock density. Figure 10 represents the projection of the variables based on their loadings on the space delimited by the first two PCs.

The first PC (PC1) explained 28.45% of the total variance (Table 5). Rain and Elev showed a high influence over the positive part of this PC1 (0.83 and 0.79, respectively), while LS-temp showed the highest negative factor loading (−0.71). A moderate negative value was also observed with DD (−0.56). Hence, PC1 shows that the main factors controlling SWB density are climate (rain and LS-temp) and topography (Elev and DD). It could be concluded that the physical characteristics of the area play a large role in the distribution of SWBs in this region.

**Table 5:** Principal component (PCA) factor loading, eigenvalue along with variance and cumulative variance

	**PC 1**	**PC 2**	**PC 3**	**PC 4**
WB	−0.17	0.60	0.52	0.11
DD	−0.56	0.34	−0.46	0.22
PD	−0.21	0.09	−0.60	0.20
LD	− 0.40	0.08	0.67	0.25
Elev	0.79	−0.33	0.18	0.01
Rain	0.83	0.37	−0.04	0.02
LS-temp	−0.71	−0.19	0.16	0.02
SPI	0.30	0.84	−0.06	0.13
TWI	−0.27	0.34	0.006	−0.86
Eigenvalue	2.56	1.61	1.37	0.93
% total variance	28.45	17.94	15.24	10.35
Cumulative variance	28.45	46.39	61.64	72.00

*WB* density of SWBs, *LD* livestock density, *DD* drainage density, *LS-temp* land surface temperature, *SPI* standardized precipitation index, *TWI* topographic wetness index, *PD* population density, *SM* soil moisture, *Elev* elevation

The second PC (PC2) explained 17.94% of the total variance and was mainly defined by the influence of SPI and WB over the density of SWBs according to the aridity of the climate.

The third component (PC3) explained 15.24% of the total variance, being well related with the PD (−0.60) and LD (0.67), highlighting the influence of socioeconomic aspects and the probable opposite role played on the construction of WBs.

The fourth component (PC4) explained 10.35% of the total variance and was strongly related with TWI, which is a useful parameter for identifying the areas where water accumulates, which probably point to the role that this aspect plays during the selection of suitable places.

## Discussion

This study focused on small ponds used for livestock purposes, for irrigation of small plots, and in some cases, for fish farming. In response to the growing demand for water and the need to improve agricultural production and to improve the livelihood of the rural population, the use of surface water, especially for SWBs, has become a common strategy (Milano et al., 2013).

WBs are crucial for the socioeconomic development of Mediterranean regions, particularly in rural areas such as Extremadura. This research confirms, on one hand, that Extremadura has a significant WBs density (2.4 WB km^−2^) and, on the other hand, that they are not randomly distributed around the region, according to the statistics provided by spatial analysis such as the nearest neighbor (Mohd Radi et al., 2018), Getis-Ord Gi* hot spot analysis (Rossi & Becker, 2019), and Moran’s index. In addition, Extremadura has a significant number of SWBs that enhance the water storage capacity of the region and play a crucial role in the socioeconomic development and the protection of the environment. According to Jlassi et al. (2016), decision makers, managers, and farmers have opted for—and based on—small ponds of water for the following reasons: (i) to increase water storage capacity in the region; (ii) to increase water pressure to allow sprinkler irrigation; and (iii) to make the water distribution process more flexible and more efficient.

Table 2 shows the predominance of ponds < 1000 m^2^ in the region, representing 85% of the total number of ponds and a clear decline in the number of ponds as their size increases. Smaller ponds are generally private efforts, representing a strategic decision of the individual farmer to solve occasional problems of water availability, especially in the summer period when rainfall is scarce, and to have a reserve of water for their uses (e.g., water supply for livestock). By comparing these small ponds with the location of the so-called large reservoirs (class 5), it can be argued that they are mainly built by regional and national institutions, for collecting water from larger rivers.

Despite Extremadura is recognized like a region of big reservoirs—very appreciated by fishing lovers—64.53% of the surface covered by water is dammed by WBs smaller than 0.01 km^2^, representing also 98.9% of the total perimeter. Comparing these values with similar studies in literature, we can observe that in Estonia (45,228 km^2^, 111,552 WBs in total) WBs < 10,000 m^2^ only represent 8.79% of the total area and 70.29% of the total perimeter, and in France (675,417 km^2^), these values reach 17.50% of the total area and 58.76% of the total perimeter (Terasmaa et al., 2019).

In Extremadura, the spatial distribution of WBs is extensive all over the region, except in the highest mountains and near the main rivers. Referring to the land use map, it can be observed that their spatial distribution does not have a favorite class or a tendency towards one direction in the area, but they cover all the study area. Class 5 represents the largest water bodies, and its abundance is mainly in the center of the study area, in the following classes, agricultural areas and forest and semi-natural areas. They are almost absent in the north of the study area, and there is some concentration towards the north west for class 3 compared to the south east. For the other classes, they are all around the area and cover the whole region.

According to Bartout et al. (2015), the origin and spatial distribution of WBs vary in accordance with geology, climate, water balance, groundwater situation, topography, altitude, economic trends, land use, sociology, historical conditions, etc. The geostatistical analysis has returned a moderate spatial dependence of WBs (Cambardella et al., 1994). Thus, the presence of spatial clusters highlighted by spatial analyses and a certain extent of spatial dependence, confirmed by geostatistical analysis, lead to conclude that some physical and/or socioeconomic reasons should explain these spatial patterns.

Bivariate and multivariate analyses—such as correlation analysis and PCA—are allowed to find out what are the variables that influence the most. In Fig. 10, a multivariate statistics (principal component analysis (PCA)) and the projection of the parameters used in this study to understand and comprehend the factors controlling and contributing to the spatial distribution of WBs show that all the parameters have an impact on the existence of the WBs, as well as on their spatial distribution—their spatial dependence is described by the variogram in the geostatistical analysis. Figure 10 clearly shows that the climatic and topographic factors have the greatest impact; anyway, not all the parameters have the same influence; rain and LS-temp give a greater contribution than SPI, despite being all included in the climatic factor, and Elev has a more important role than DD, even if both are part of the topographic factor. The socioeconomic factor, represented by the PD and the LD, is not to be overlooked.

The multivariate analysis reveals that a thorough examination of all the topographical, climatic, and socioeconomic conditions of surface WBs is necessary to preserve biodiversity and accompany farmers in their projects, providing favorable conditions for regional and national development.

The increasing urbanization of society and the expansion of agriculture and commercial mining activities have led to an increase in the number of small artificial WBs in many parts of the world (Grinham et al., 2018). This increase was not random but is controlled by parameters that allowed the existence or creation of new WBs to satisfy water needs. Livestock and population density, climate, topography, and the willingness of local authorities and farmers are the most influential factors, as demonstrated in multivariate statistical studies. The PCA showed that more than two-thirds of the total information (72%) is explained by the first four components. The expansion of agriculture through the introduction of agroindustry and the rapid rate of population growth are leading local and national decision makers to build WBs in natural areas that favor water harvesting. Small ponds play also a role in hydrological regulation, elimination of nutrients, fish production, recreation, and providing refuge for wildlife (Céréghino et al., 2007; Kristensen & Globevnik, 2014).

Small ponds contribute to improve water management by minimizing water loss and waste, and by giving more flexibility to the irrigation system, and contribute to the farmers’ revenue by improving the quality of their work and allowing them to expand their enterprise creating also new jobs. Due to their characteristics, SWBs are among the most valuable and potentially the easiest mean to preserve the regional aquatic biodiversity. Ponds typically outnumber larger lakes by a ratio of about 100:1 (Oertli et al., 2005); recent research has highlighted their significance for the conservation of biodiversity (Scheffer et al., 2006) because, despite of their size, they disproportionately contribute to regional biodiversity, for example, when compared to streams, large rivers, or lakes (Williams et al., 2004). Thus, ponds pose a challenge to traditional biology conservation methods, which have focused mostly on large-scale ecosystems (Meffe & Carroll, 1997). They have also the benefit of creating a local microclimate. Nevertheless, evaporation in small ponds can represent a relatively large volume of water loss. Jlassi et al. (2016) estimated that in the Aragon area in the north east of Spain, the loss is only 4.9% of the total storage capacity, although it is expected to increase in the near future given the current annual temperature trend and could then reach about 7.5% of the total storage capacity.

Extremadura’s overburdened population, combined with extensive agricultural practices and animal husbandry waste, necessitates highly efficient drainage systems. The government and decision-makers, as well as farmers, are accountable for these drainage facilities, which protect WBs from pollution—which has a direct detrimental impact on human’s and animal’s health. The infiltration of certain volumes of water from these WBs into the soil can contaminate the water table and the deepest aquifers, which are used for agricultural and industrial purposes, and impact soil quality and productivity, resulting in infertile land. Protecting WBs against pollution is essential to guarantee the socioeconomic development of the region.

Since these surface waters could be polluted, affecting soil quality and livestock health, these aspects should be investigated in future work.

It is very important to understand the role of small ponds in Extremadura as a solution to store surface water during the rainy season, to compensate the lack of rainfall and evaporation losses during dry periods (Pulido et al., 2020). They are mainly used for agricultural purposes (livestock consumption and crop irrigation), but there is a wide list of functions that they can perform (fish farming, recreational use, ecotourism, amphibian preservation, etc.). This large number of WBs can be used for satisfying crop requirements or converting rainfed fields into irrigation agriculture in case of food scarcity risk. Fishing is one of the most attractive recreational purposes for stakeholders (personal communication with the regional director of the fishing service) as a way of entertainment of local people. Nonetheless, the essence of WBs in Extremadura is provided by watering ponds (*charcas* in Spanish) that are used for drinking livestock in the extensive rangelands and grasslands, the dominant land use in the region.

## Conclusion

All the WBs existing in Extremadura were gathered and analyzed, finding that (i) the great importance of SWBs, despite Extremadura, is recognizable by its big reservoirs, and ii) the spatial dependence of these reservoirs explains why some areas of the region have a high density of WBs and other areas have not. In addition, these spatial patterns of concentration have been confirmed by several techniques of spatial analysis. The extensive livestock husbandry as dominant land use, expressed as livestock density, seemed to be the most influencing variable on WBs density. Thus, the main reason behind the large number of existing WBs in Extremadura has been the human necessity of guaranteeing water for livestock in summer. Nonetheless, further research focused on water quality and efficiency of WBs under pessimistic climate models is still needed, to properly understand how long the endangered traditional land systems such as the Iberian *dehesas* and *montados* could be effective.

## Data Availability

The datasets generated during and/or analyzed during the current study are available from the corresponding author on reasonable request. Data are available on request from the authors.
